# Interplay of *Helicobacter pylori*, fibroblasts, and cancer cells induces fibroblast activation and serpin E1 expression by cancer cells to promote gastric tumorigenesis

**DOI:** 10.1186/s12967-022-03537-x

**Published:** 2022-07-21

**Authors:** Xueshu Chen, Wei Chen, Yan Zhao, Qinrong Wang, Wenling Wang, Yining Xiang, Hang Yuan, Yuan Xie, Jianjiang Zhou

**Affiliations:** 1 Key Laboratory of Endemic and Ethnic Diseases, Ministry of Education & Key Laboratory of Medical Molecular Biology of Guizhou Province, Guizhou Medical University, Guiyang, China; 2 Department of Laboratory Medicine, Guizhou Cancer Hospital, Guiyang, China; 3 Department of Hematology, Affiliated Hospital of Guizhou Medical University, Guiyang, China; 4 Department of Abdominal Oncology, Guizhou Cancer Hospital, Guiyang, China; 5 Department of Pathology, Affiliated Hospital of Guizhou Medical University, Guiyang, China

**Keywords:** *Helicobacter pylori*, Fibroblasts, Gastric cancer, Serpin E1, RNA-sequencing

## Abstract

**Background:**

*Helicobacter pylori* (*H. pylori*) can disrupt the tight junctions between gastric epithelial cells and penetrate the intercellular spaces acting on epithelial cells, normal fibroblasts (NFs), and cancer-associated fibroblasts (CAFs), but their interaction in gastric cancer tumorigenesis and progression remains unclear.

**Methods:**

Primary CAFs and NFs were isolated from paired gastric cancer tissues and adjacent normal tissues and identified by immunofluorescence staining and western blot analysis for FSP-1, α-SMA, FAP, and vimentin expression. RNA-sequencing was used to compare the transcriptomes between CAFs and NFs. The expressions of FAP, lumican, and α-SMA, human cytokine array, and Transwell assay were used to assess the transformation of NFs to CAFs. CCK-8 assay, colony formation, flow cytometry, Transwell assay, and nude mouse xenograft model were used to determine the effects of Serpin E1 on cell proliferation and metastasis in vitro and in vivo. Finally, Serpin E1 and/or FAP expression was measured in *H. pylori*-infected gerbil gastric mucosa and human gastric cancer tissues.

**Results:**

Gastric CAFs are inflammatory CAFs with α-SMA^low^FAP^high^lumican^high^. The interplay of *H. pylori*, fibroblasts, and cancer cells promotes the transition of NFs to CAFs by inducing cytokine release, especially Serpin E1. Long-term *H. pylori* infection and CAFs induce Serpin E1 expression in gerbil gastric tissues and human gastric cancer cells. Serpin E1 overexpression enhances the growth, migration, invasion of gastric cancer cells in vitro, and xenograft tumor growth in nude mice via inducing angiogenesis. Serpin E1 and FAP were highly expressed in cancer cells and CAFs of gastric cancer tissues, respectively, and a good correlation was observed between their expression. Higher Serpin E1 expression is negatively associated with the overall survival of patients with gastric cancer.

**Conclusions:**

The interplay of *H. pylori*, fibroblasts, and cancer cells induced Serpin E1 expression to promote the activation of NFs to CAFs and gastric carcinogenesis. Targeting Serpin E1 will provide a promising therapeutic strategy for gastric cancer by disrupting the interaction between *H. pylori*, CAFs, and gastric cancer cells.

**Supplementary Information:**

The online version contains supplementary material available at 10.1186/s12967-022-03537-x.

## Introduction

Gastric cancer is the fifth most common cancer and the fourth leading cause of cancer-related death worldwide [1]. Although the incidence of gastric cancer has declined over the past 50 years globally, it is still a considerable health burden in developing counties. The global 5-years survival rates of gastric cancer patients remain unsatisfactory [2–4]. Most patients are diagnosed with an advanced stage of gastric cancer, and the recurrences and metastases of patients are frequently observed [5]. The most important risk factor for gastric cancer is *Helicobacter pylori* (*H. pylori*), classified as a class I carcinogen for gastric cancer by IARC in 1994 [6]. Although numerous studies have been carried out on the association between *H. pylori* infection and gastric cancer, the mechanism underlying their relationship remains unclear [7–9]. In recent years, *H. pylori* have been found to disrupt the tight junctions between gastric epithelial cells and penetrate the deeper intercellular spaces to colonize the gastric epithelial cells, intercellular spaces, and the underlying lamina propria [10]. *H. pylori* at these sites, directly and indirectly, interact with fibroblasts, connective tissue, and other extracellular matrix components. Recently, the direct interaction between *H. pylori* and rat stomach fibroblasts has been proposed [11–13].

The tumor microenvironment (TME) favors the growth and metastasis of cancer cells in many solid tumors. Fibroblasts, especially cancer-associated fibroblasts (CAFs), are a critical stroma component and probably contribute to tumor initiation and progression via direct contact with cancer cells or paracrine manner [14, 15]. CAFs are a functionally heterogeneous population composed of tumor-promoting CAFs, tumor-restraining CAFs, and quiescent CAFs with different marker expressions [16]. CAFs can be identified through a series of markers such as fibroblast-activating protein (FAP), alpha-smooth muscle actin (α-SMA), fibroblast specific protein 1 (FSP-1), and vimentin [17]. Based on these markers, Ohlund and colleagues identified two distinct subtypes of CAFs in human pancreatic cancer: α-SMA^hi^IL-6^low^ myofibroblastic CAFs and α-SMA^low^IL-6^hi^ inflammatory CAFs [18]. The heterogeneity of CAFs is further reflected in its origin of several cell types, including normal resident fibroblasts (NFs), bone marrow mesenchymal stem cells, pericytes, epithelial cells, and endothelial cells [19, 20]. Among these cells, tissue-resident NFs activation in response to numerous stimuli from the TME is the primary source of CAFs and exists before tumorigenesis [21]. Moreover, the transition of NFs to CAFs can induce gene expression and phenotype changes between them [22]. However, the multifaceted interplay between *H. pylori*-induced inflammation, fibroblasts, and cancer cells are poorly understood in gastric tumorigenesis.

Serpin E1, also known as plasminogen activator inhibitor-1, is a structurally well-studied serine protease inhibitor that acts in diverse pathologies such as thrombosis and fibrosis by inhibition of urokinase-type plasminogen activators and tissue-type plasminogen activators. Subsequent studies suggest a pro-tumorigenic role of Serpin E1 [23]. Multiple cells, including tumor and stromal cells such as endothelial cells, fibroblasts, macrophage cells, and adipocytes, produce Serpin E1 [24]. Its expression is also regulated by various growth factors, cytokines, and chemokines [25]. However, Serpin E1 expression in gastric cancer cells and its interaction with *H. pylori* infection and fibroblasts remain unknown.

In the current study, we obtain the transcriptome expression profiles of CAFs and NFs isolated from paired gastric cancer and adjacent normal tissues and demonstrate the interplay of *H. pylori*, fibroblasts, and gastric cancer cells to promote the conversion of NFs to CAFs via cytokine release, especially Serpin E1. *H. pylori* and CAFs induce gastric epithelial and cancer cells to express Serpin E1, leading to cell migration, invasion, and tumor formation in vitro and in vivo. Our findings thus offer a novel insight into gastric cancer tumorigenesis during *H. pylori* infection. Targeting Serpin E1 can disrupt the interaction of *H. pylori*, CAFs, and cancer cells to provide a promising target for gastric cancer therapy, which will become an attractive option for future research.

## Materials and methods

### *H. pylori* strain and cell culture

*H. pylori* strain GZ7 (GenBank accession ID: KR154737.1) was isolated from clinical gastric cancer tissue with informed consent and the approval of the Ethics Committee of Guizhou Medical University and confirmed as a typical East Asian strain (cagA^+^) by sequencing in our previous research. Its whole-genome sequence has been submitted to the National Microbiology Science Data Center, China (Accession ID: NMDC60014578, https://nmdc.cn/). This bacterium was grown on a Columbia blood agar plate (Oxoid Ltd, Cambridge, UK) containing 10% sheep blood and 100 U/ml *H. pylori* selective supplement (Oxoid Ltd, Cambridge, UK) at 37 °C under microaerobic conditions (5% O_2_, 10% CO_2_, 85% N_2_).

Human gastric cancer cell lines AGS cells (ATCC, VA, USA), MNK45 cells, HUVECs (CBCAS, Shanghai, China), and primary GC cells (isolated from gastric cancer tissues in our previous study) were cultured in RPMI-1640 medium (Hyclone, Logan, UT, USA) containing 10% fetal calf serum (Gibco, Carlsbad, CA, USA) and incubated in a humidified incubator with 5% CO_2_ at 37˚C.

### Samples of human gastric cancer tissues

Twelve pairs of clinical gastric cancer and para-cancer normal tissues (at least 5 cm from the outer margin) from the surgical resection were collected at the Affiliated Hospital of Guizhou Medical University, China, from January 2020 to December 2021. Half of the tissues were used for pathological analysis, and the other half were transferred to the laboratory for IHC analysis within two hours. The diagnoses were confirmed by two pathologists. Ten patients were male, and two were female. Their median age was 60 (range 27–86) years. Eight cancers were adenocarcinoma, and four were the diffuse type. Nine cancers occurred in the gastric corpus, and three were in the gastric antrum. All patients were positive for the C^13^ urea breath test (a diagnosis method of *H. pylor*i infection). All subjects gave their informed consent for inclusion before participating in the study. The study was conducted following the Declaration of Helsinki, and the protocol was approved by the Ethics Committee of Guiyang Medical University (Approved number: 2017(43)).

### Stomach tissue sections of Mongolian gerbils infected with *H. pylori*

In our previous study, *H. pylori* NCTC 11637 (ATCC 43504, *cagA*-positive) was used to infect intragastrically Mongolian gerbils for 24 months to establish *H. pylori*-related gastric disease models successfully. In these gerbils, chronic superficial gastritis, erosive gastritis, atrophic gastritis, intestinal metaplasia, and well-differentiated gastric cancer were pathologically observed at 3, 6, 12, and 24 months after *H. pylori* infection [26]. At different time points, the gerbils (3 gerbils per time point) were sacrificed after anesthesia, and their stomach tissues were removed, fixed, and embedded in paraffin. In the present study, the paraffin-embedded gerbil stomach tissues were cut into 5-μm sections for IHC staining.

### Isolation and culture of primary fibroblasts

Three paired primary fibroblasts (CAFs and NFs) were obtained from three patients with poorly differentiated gastric adenocarcinomas (ages: 52–54 years) undergoing cancer resection at the Affiliated Hospital of Guizhou Medical University, China. The CAFs and NFs were isolated from cancer tissue and matched normal tissue at least 5 cm from the outer tumor margin [27]. Fresh samples were washed with RPMI-1640 medium containing 100 U/ml penicillin and 100 μg/ml streptomycin (Hyclone, Logan, UT, USA).), cut into small pieces, and incubated in a 5 ml solution containing 2 mg/mL collagenase IV (Sigma-Aldrich, MO, USA) for 1.5–2.5 h at 37 °C. The digested cells were passed through a 200-mesh cell sieve and centrifuged at 1500 rpm for 10 min. The single-cell suspension was cultured in a fibroblast medium (ScienCell, CA, USA) consisting of 2% fetal bovine serum, 1% fibroblast growth supplement, and 1% penicillin/streptomycin. After that, the suspension was incubated in a humidified incubator with 5% CO_2_ at 37˚C for 48 h, allowing fibroblasts to attach to the culture plate. The adherent cells were further passaged and cultured. CAFs and NFs from the 3rd to 5th passages were used for our experiments. Three subjects gave their informed consent, and the protocol was approved by the Ethics Committee of Guiyang Medical University [No: 2017(43)].

### RNA sequencing (RNA-seq) analysis

Total RNA was isolated from three paired primary CAFs and NFs and reversely transcribed into cDNA to generate an indexed Illumina library, then sequenced at the GENEWIZ Biotechnology Co., Ltd. (Suzhou, China) using Illumina HiSeq 2000 platform. After normalizing the expression of genes, the differentially expression genes (DEGs) with fold-change > 2 and FDR < 0.05 were screened out by comparing the Fragments Per Kilobase per Million (FPKM) between CAFs and NFs using an online analysis tool (http://www.omicsbean.cn/). The DEGs common to the three pairs of CAFs and NFs were selected for follow-up bioinformatic analysis. A heatmap of the DEGs was generated by the online tool (http://www.omicsbean.cn/). Subsequently, the DEGs were analyzed by Gene Ontology (GO) using the Gene Ontology Consortium (http://geneontology.org/), Kyoto Encyclopedia of Genes and Genomes (KEGG) pathways using KOBAS (http://kobas.cbi.pku.edu.cn/), and the protein–protein interaction (PPI) networks using the STRING tool (https://string-db.org/). Cytoscape software (version 3.7.2) was used to visualize KEGG pathways and PPI networks. Gene set enrichment analyses were performed by Gene Set Enrichment Analysis (GSEA) (v4.1.0) based on the gene expression (FPKM). NOM p-value < 0.01 and FDR q-value < 0.25 were considered as significant gene set.

### Human cytokine array

NF and AGS cells were infected with *H. pylori* for six hours at an MOI of 50, and then *H. pylori* were removed. The culture supernatants were collected after another seven-day culture. The cytokine levels in the supernatants were determined by a Proteome Profiler Human Cytokine Array Kit (ARY005B, R&D Systems, MN, USA) according to the manufacturer's instructions. Briefly, membranes were incubated with blocking buffer at room temperature for one hour. Simultaneously, the cell supernatants (0.5 ml) were mixed with the biotinylated detection antibody cocktail (15 μl) at room temperature for one hour, and then the mixture was incubated with the membrane overnight at 4 °C. Afterward, the arrays (membranes) were incubated with horseradish peroxidase-conjugated streptavidin for 30 min at room temperature. The arrays subsequently were exposed to ECL substrate (Amersham, Bucks, UK), and the images were obtained using ECL chemiluminescence. The array images were analyzed with Image J. Cytokine levels are expressed as the percentage of the average density of two Cytokine Spots divided by the average density of six Reference Spots. Proteome Profiler Human Cytokine Array list was shown in Additional file 1: Table S1.

### Direct and transwell co-culture models

NFs or CAFs and AGS cells were co-incubated with *H. pylori* in a 6-well plate for 6 h at an MOI of 50, and then free *H. pylori* were removed via extensive washing with PBS to construct the direct co-culture models. NFs or CAFs were seeded into the lower chamber, and *H. pylori* and/or AGS cells were added to the upper chamber of the Transwell (0.4-μm pores). After the cultures were incubated for six hours, *H. pylori* were removed via washing with PBS to construct the Transwell co-culture models. The co-cultures in the two models were further cultured for 72 h and seven days for in vitro experiments.

### Lentivirus infection

A lentiviral expression vector containing cDNA to code Serpin E1 and control lentiviral were acquired from Jikai Co. (Shanghai, China) and were used to infect AGS, MNK45, and primary GC cells using Lipofectamine 2000 (Invitrogen, CA, USA). Subsequently, three stable cell lines overexpressing Serpin E1 or empty vector were generated by puromycin selection and used for in vitro experiments.

### Cell viability assay

Cell viability was evaluated by the Cell Counting Kit (CCK-8) (Dojindo, Kumamoto, Japan). In brief, CAFs or NFs were seeded in a 96-well plate at a density of 1000 cells /well and cultured for six days. Similarly, AGS cells with Serpin E1 overexpression or empty vector were also seeded in a 96-well plate at a density of 500 cells /well and cultured for six days. Then, 10 μl of CCK-8 solution were added into the culture medium in each well on days 1, 2, 3, 4, 5, and 6 of culture, respectively. After incubation for two hours, the absorbance at 450 nm was measured by a microplate reader. The growth curve of fibroblasts and AGS cells was plotted based on the *A*_450_ values. Data presented as mean ± SD (n = 6 per group).

### Colony formation assay

NFs or CAFs were co-cultured with AGS cells for ten days in a 24-well plate at a ratio of 10:1 (NFs/CAFs: AGS cells). Similarly, AGS cells with Serpin E1 overexpression or empty vector were seeded in a 24-well plate for ten days. The medium was removed, and the colonies were washed gently with PBS, fixed in 4% paraformaldehyde for 30 min, and stained with 0.1% crystal violet for 30 min. The colonies were counted under a microscope.

### Cell cycle assay

AGS cells were collected, washed with cold PBS, and then fixed in cold 70% ethanol overnight at 4 °C. The next day, the cells were centrifugated at 1000 rpm for 5 min and resuspended in 450 μl 1 × PBS containing 50 μl RNase A. After incubation at 37 °C for 30 min, the cells were stained with 10 μl Propidium Iodide (50 μg/ml, BD Biosciences, San Jose, CA) for another 30 min away from light. Cell cycle distribution was determined by flow cytometry, and the results were analyzed with the FloJo software.

### Apoptosis assay

Three stable cell lines with Serpin E1 overexpression or empty vector were cultured overnight in a serum-free medium to synchronize the cell cycle. The medium was replaced with a complete medium containing arsenic trioxide (As_2_O_3_, 20 and 40 μM, Sigma-Aldrich, MO, USA) for 24 h. Then, 10^5^ cells were resuspended in 500 μl of 1 × binding buffer and stained with 5 μl of Annexin V-APC and 10 μl of 7-AAD (Biosciences, San Jose, CA) for 10 min, protected from light. The cell suspension was filtered through a nylon mesh (400 mesh), and cell apoptosis was detected by flow cytometry. Data were analyzed with the FloJo software.

### Transwell migration and Matrigel invasion assay

Transwell assay was performed using an 8 μm pore size 24-well Transwell Chambers (Costar, Cambridge, MA, USA) without and with Matrigel (Biosciences, San Jose, CA). AGS cells or NFs/CAFs in 6-well plates were infected with *H. pylori* for six hours at an MOI of 50, and then *H. pylori* were then removed. After the cultures continued for seven days, 1 × 10^4^ AGS cells in 200 μl of 1% FBS medium were seeded into the upper chamber, and 5 × 10^4^ NFs or CAFs in 800 μl medium containing 10% FBS were seeded into the lower chambers. After 24 h (migration assay) and 72 h (invasion assay), the Transwell inserts were moved out, fixed in 4% paraformaldehyde for 30 min, and stained with 0.1% crystal violet for 30 min. The non-migrated or invaded cells were removed from the upper surface of the chambers. The number of migrated and invaded cells was counted under a microscope.

For stably transfected cancer cell lines, 10^4^ cells were seeded in the upper chamber in a 1% FBS medium, and an 800 μl medium containing 10% FBS was added into the lower chambers. After 24 h (migration assay) and 48 h (Matrigel invasion assay) incubation, the Transwell inserts were moved out for follow-up experiments according to the method described above.

### Immunocytochemistry, immunohistochemistry, and immunofluorescence

The cell climbing slices, including CAFs, NFs, AGS cells, AGS cells co-cultured with CAFs for three days at a 1:1 ratio, and AGS cells infected with *H. pylori* for seven days at an MOI of 50, were fixed in 4% paraformaldehyde for 30 min at room temperature. After washing with PBS, the slices were permeabilized with 0.5% Triton X-100 for 30 min and blocked with 5% bovine serum albumin for 60 min. The slices were incubated with different primary antibodies in a humid box overnight at 4 °C and then corresponding HRP-conjugated secondary antibodies (CST, MA, USA) for two hours at room temperature. Subsequently, a DAB reaction was performed using a DAB substrate kit (Abcam, Cambridge, UK), and the reaction was terminated by adding a 30% H_2_O_2_ solution. DAB-visualized slices were counter-stained successively with hematoxylin, 1% acid alcohol, and ammonium water. Finally, the slices were dehydrated in graded ethanol, followed by clearing in xylene. Images were acquired microscopically.

For immunohistochemistry, the tissue sections, including gerbil stomach tissues, tumor tissues of mouse xenografts, and human gastric cancer tissues, were deparaffinized in xylene and rehydrated in ethanol with an increased concentration. Then, antigens were retrieved using high pression in 0.01 M citrate buffer (pH 6.0), and the endogenous peroxidases were blocked using 3% H_2_O_2_. After that, the sections were incubated with primary and secondary antibodies. The following experiments were performed by the same methods as mentioned above. The quantification of staining density was analyzed using IHC Profiler from Image J software, and the IHC scores were calculated as follows: IHC scores = intensity score × percentage score [28].

For immunofluorescence, a fluorescent secondary antibody was incubated with the slices in a humid box at room temperature for one hour. Cell nuclei were counter-stained with DAPI (Sigma-Aldrich, MO, USA). Images were acquired on a confocal microscope (Olympus, Japan).

The following primary antibodies were used: rabbit polyclonal anti-vimentin, anti-FSP1, anti-Serpin E1, anti-CD31, anti-Ki67, and anti-*H. pylori* antibodies (Abcam, Cambridge, UK), rabbit polyclonal anti-α-SMA antibody (Proteintech, Chicago, USA), rabbit polyclonal anti-FAP antibody (GeneTex Inc., CA, USA).

### Western blotting assay

Cells were lysed in RIPA buffer containing a complete protease and phosphatase inhibitor cocktail (Sigma-Aldrich, MO, USA). The protein concentration of the cell lysates was quantified by a BCA Protein Assay Kit (Pierce, Rockford, IL, USA). The same protein samples were resolved onto 10% SDS-PAGE and then transferred onto PVDF membranes (Millipore Q, Billerica, MA, USA). After blocking with 5% nonfat milk at 37℃ for two hours, the membranes were incubated with primary antibodies overnight at 4℃, followed by incubation with the HRP-conjugated secondary antibody for two hours at room temperature. GAPDH antibody was used as a loading control. Finally, the protein band images were captured by a Gene Detection System with an ECL reagent (Thermo Fisher Scientific, MA, USA). The primary antibodies used in the experiments were rabbit polyclonal anti-FAP antibody (GeneTex Inc., CA, USA), rabbit polyclonal anti-lumican antibodies (Absin, Shanghai, China), rabbit polyclonal anti-Serpin E1, anti-Vimentin, and anti-Urease B antibody (Abcam, Cambridge, UK), rabbit monoclonal anti-GAPDH antibody (CST, MA, USA), and rabbit polyclonal anti-α-SMA antibody (Proteintech, Chicago, USA).

### Enzyme-linked immunosorbent assay (ELISA)

ELISA kit of Serpin E1 (Invitrogen, CA, USA) was used to detect Serpin E1 levels in cell culture supernatants according to the manufacturer's instructions. The experiment was repeated three times.

### Tube formation assay

Matrigel was melted at 4℃ overnight and diluted with serum-free medium (1:4). After Matrigel polymerization, human umbilical vascular endothelial cells (HUVECs, 5 × 10^4^) were suspended in a medium and seeded on the Matrigel in the 24-well plate or the lower chamber of Transwell. Human recombinant Serpin E1 (PeproTech, USA) was added to the 24-well plate at a concentration of 2 ng/ml, or 1 × 10^5^ cells, including AGS, MNK45, and primary gastric cancer cells overexpressing Serpin E1 and control vector, were added to the upper chamber of Transwell. After culturing for 24 h, tube formation of HUVECs was observed and photographed.

### Tumor xenograft assay

Ten six-week-old male BALB/c nude mice were purchased from Chongqing Tengxin Biotechnology Co., LTD. (Chongqing, China) and raised under specific pathogen-free conditions at the Animal Center of Guizhou Medical University. 1 × 10^7^ primary GC cells with stable Serpin E1 overexpression or empty vector were suspended in 200 µl PBS and injected subcutaneously into the right flanks of each mouse. Tumor size was monitored once every two days for 24 days. The tumor volume (V) was calculated by the formula: V = 1/2 × length × width^2^ (mm3). Twenty-four days later, all mice were sacrificed after anesthesia, and the tumor tissues were stripped, weighted, and fixed in 4% paraformaldehyde for further IHC staining. The animal study was approved by the Animal Care Welfare Committee of Guizhou Medical University (Approved number:1702155).

### Analysis of The Cancer Genome Atlas (TCGA) dataset for Serpin E1

The human gastric cancer genomics dataset and the related information of patients, including 408 tumors and 211 normal tissues, were downloaded from The Cancer Genome Atlas-Stomach Adenocarcinoma (TCGA-STAD) database (https://gdc-portal.nci.nih.gov/projects/TCGA-STAD) and analyzed for Serpin E1 mRNA expression.

### Statistical analysis

All statistical analysis was performed using SPSS 22.0 (SPSS, Inc). GraphPad Prism 7 was used to generate figures. Two groups were compared using a two-tailed paired or unpaired Student’s t-test. More than two groups were compared using one-way ANOVA or two-way ANOVA. Spearman correlation analysis was used to analyze the association between Serpin E1 and FAP. The images shown are representative of at least three independent experiments. All data are presented as means ± standard deviation (SD) from at least three independent samples or experiments. A *P* value < 0.05 was considered statistically significant.

## Results

### Identification of primary fibroblasts isolated from human gastric cancer tissues

**Fig. 1:**
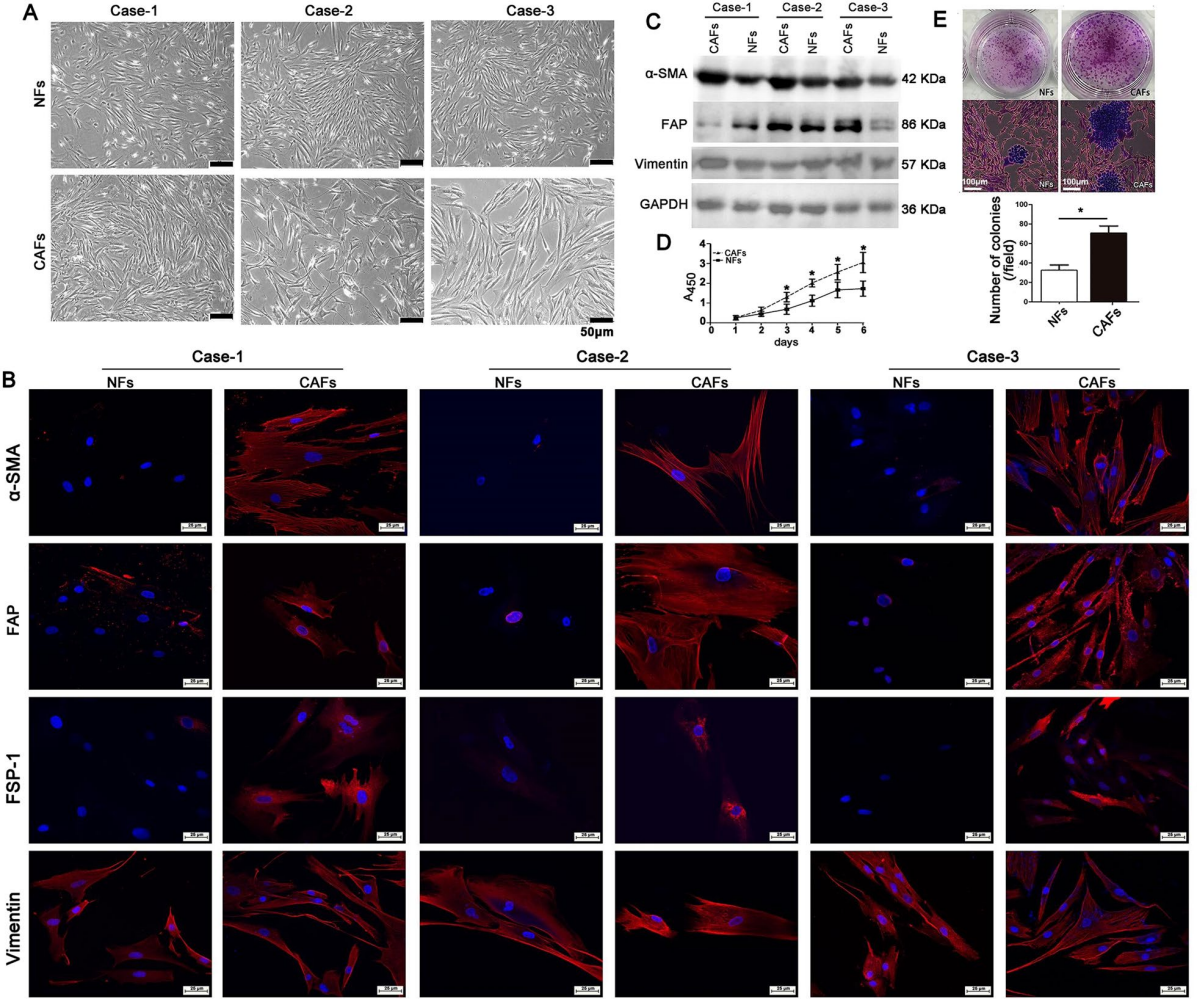
Identification of CAFs and NFs. **A** Morphology of CAFs and NFs. Scale bar = 50 μm. **B** Immunofluorescence staining for α-SMA, FAP, FSP-1, and Vimentin in CAFs and NFs. Vimentin, a specific marker of stroma cells, was used as a control. Scale bar = 25 μm. **C** Western blot analysis shows the higher expression of α-SMA and FAP in CAFs than in NFs. **D** Growth curve of CAFs and NFs was performed with CCK-8 assay at indicated time points. Each plotted data point represents the mean ± SD (n = 6). *P < 0.05. **E** Colony formation of AGS cells co-cultured with CAFs or NFs for ten days at CAFs/NFs: AGS cells ratio of 10:1 (upper panel) and the morphology of the co-cultured AGS cells and CAFs under a light microscope (middle panel). The bar graph shows the quantification of colony number (lower panel). The data are shown as mean ± SD (n = 3). Scale bar = 100 μm. *P < 0.05

Three pairs of CAFs and NFs were isolated from cancer tissues and adjacent normal tissues of three patients with gastric adenocarcinoma. Although both CAFs and NFs display elongated spindle-shaped morphology, CAFs have larger and longer cell bodies that form an intercellular connection and slightly disordered arrangements than NFs (Fig. 1A). Immunofluorescence staining showed that α-SMA, FAP, and FSP-1 were expressed at higher levels in CAFs than in NFs, while vimentin, a specific marker of stroma cells, was equally expressed in CAFs and NFs (Fig. 1B), indicating that CAFs were the activated fibroblasts. Western blot analysis further confirmed the high expression of α-SMA and FAP in CAFs except for FAP expression in Case 1 (Fig. 1C). Furthermore, the growth of CAFs was significantly faster than that of NFs (Fig. 1D). When CAFs or NFs were co-cultured with human gastric cancer cell line AGS cells, CAFs could significantly facilitate the colony formation of AGS cells compared to NFs (Fig. 1E), suggesting the tumor-promotion effects of CAFs in vitro.

### Analysis of differentially expressed genes between CAFs and NFs

**Fig. 2:**
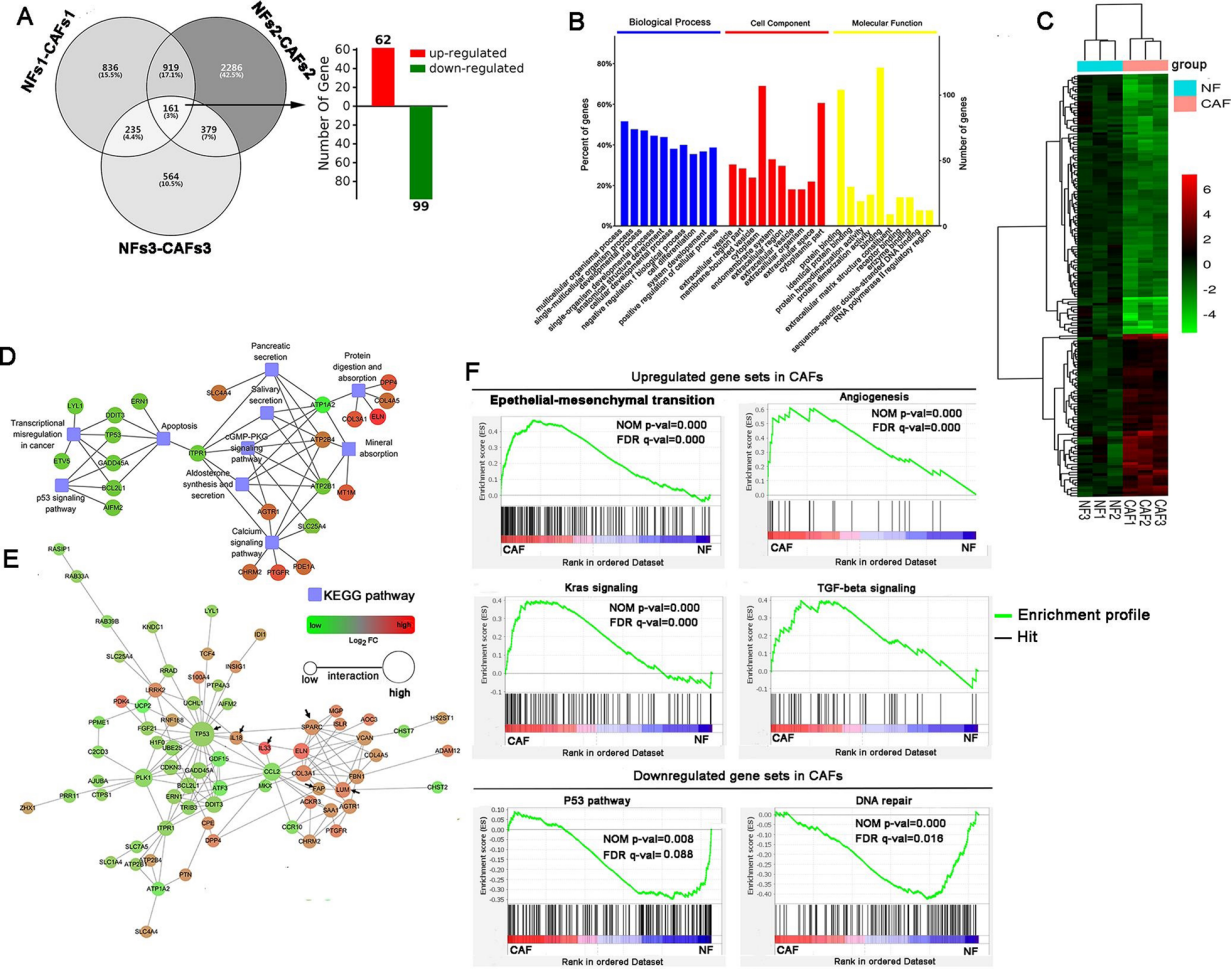
Transcription profiles between NFs and CAFs by RNA-seq analysis. **A** Venn diagram and bar plot show the shared 161 DEGs between three-pared CAFs and NFs isolated from cancer tissues and adjacent normal tissues of three patients with gastric adenocarcinoma. **B** Top 10 GO enrichment analyses of 161 DEGs. **C** Heatmap depicts the results of hierarchical clustering analysis based on 161 DEGs between CAFs and NFs. **D** KEGG pathway enrichment analyses of 161 DEGs. **E** PPI analysis reveals that p53-associated proteins are down-regulated, while SPARC-associated proteins are up-regulated in CAFs compared to NFs. **F** Up-regulated pathways and down-regulated pathways in the GESA

By RNA-seq analysis, we quantified 161 DEGs common to three-pair CAFs and NFs, including upregulation of 62 genes and downregulation of 99 genes in CAFs compared to NFs (Fig. 2A). The data were further confirmed by heatmap (Fig. 2C). The top 10 GO terms of the 161 DEGs were distinctly associated with developmental processes, cell differentiation, protein–protein binding, and extracellular matrix structural constituents and were primarily located in the extracellular region and vesicle (Fig. 2B). The KEGG pathway analysis indicated that the p53 signaling pathway, apoptosis, and transcriptional misregulation in cancer were uniquely enriched by the downregulated proteins, while the calcium signaling pathway and protein digestion and absorption were uniquely enriched by the upregulated proteins in CAFs (Fig. 2D). The PPI analysis further verified the downregulation of P53 and P53 pathway-related proteins in CAFs (Fig. 2E). Moreover, the high expression of FAP, Lumican, IL18, IL33, and secreted protein acidic and rich in cysteine (SPARC involved in epithelial-mesenchymal transition) in CAFs was observed by PPI analysis. GSEA analysis revealed that the gene sets of the epithelial-mesenchymal transition, angiogenesis, Kras signaling, and TGF-beta signaling were enriched in CAFs, while the gene sets of the P53 pathway and DNA repair were enriched in NFs (Fig. 2F). These data suggested that CAFs favored the nutrition supply and metastasis of cancer cells. The details of the 161 DEGs are listed in Additional file 1: Table S2.

### Interplay of *H. pylori*, NFs, and cancer cells induced cytokines production to promote the functional conversion of NFs into CAFs

**Fig. 3:**
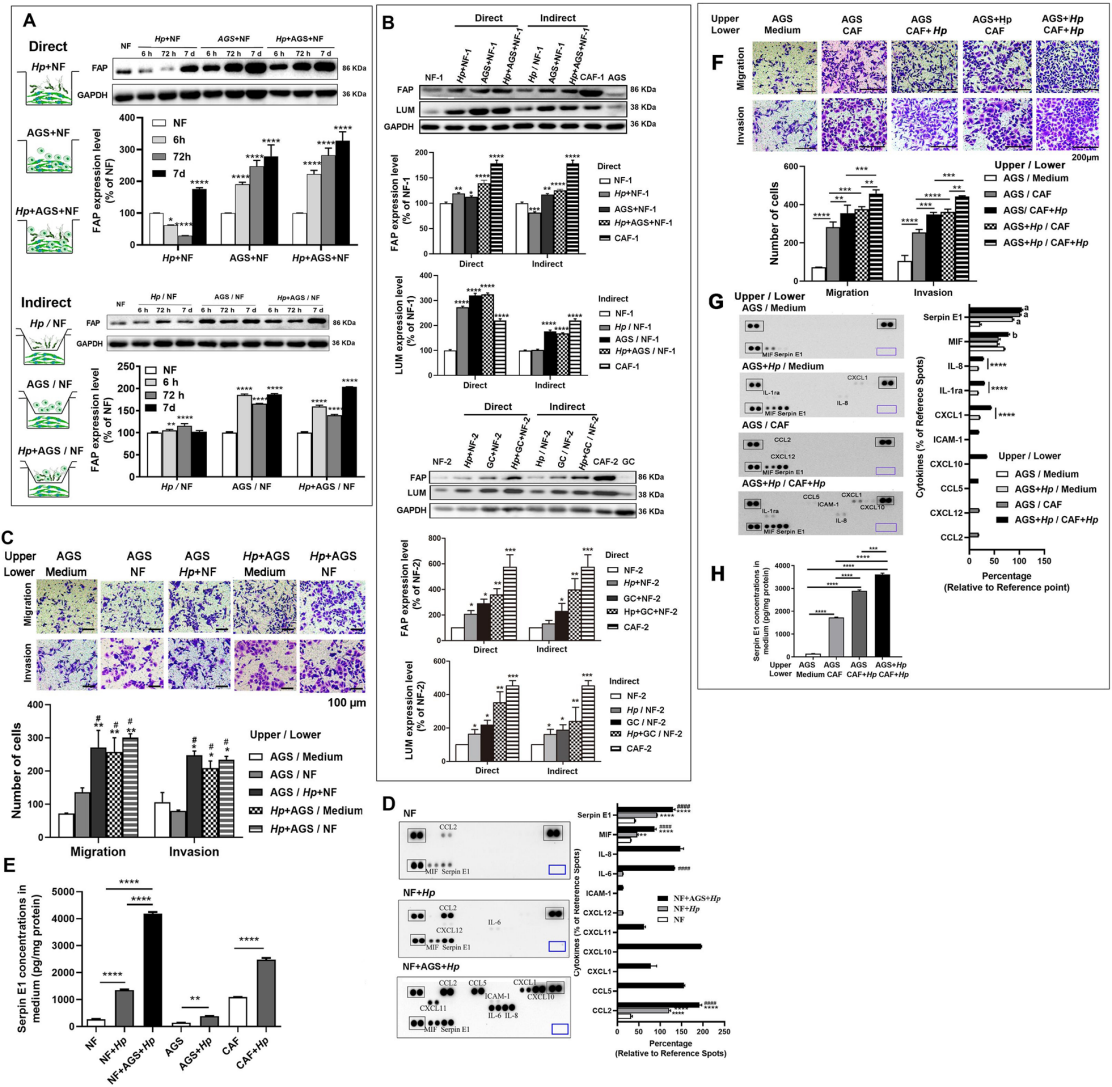
Co-culture of *H. pylori* (*Hp*), NFs, and gastric cancer cells induces cytokines release to promote NFs activation. **A** Western blot shows FAP expression in the direct and Transwell (indirect) co-culture of *H. pylori*, NFs, and AGS cells for the indicated time at a multiplicity of infection (MOI) of 50. The bar graph shows the quantitation of FAP levels. *P < 0.05, **P < 0.01, and ****P < 0.0001 vs. NF. **B** Western blot shows FAP and Lumican (LUM) expression in the direct and Transwell co-culture of *H. pylori*, NFs, and AGS or primary GC cells (GC) for 7 days at an MOI of 50. The bar graph shows the quantitation of FAP and LUM levels. *P < 0.05, **P < 0.01, ***P < 0.001, and ****P < 0.0001 vs. NF. **C** Transwell migration and Matrigel invasion assays in the co-culture of *H. pylori*, NFs, and AGS cells for 7 days at an MOI of 50. The bar graph shows the quantification of migrated or invaded cells. *P < 0.05 and **P < 0.01 vs. AGS/medium. ^#^*P* < 0.01 vs. AGS/NF. **D** Cytokine detection in the culture or co-culture supernatants of *H. pylori*, NFs, and AGS cells for 7 days using a human cytokine array. Black rectangles indicate Reference Spots. Blue rectangles indicate the negative control. The bar graph shows the quantification of cytokines, expressed as the average density of two cytokine spots divided by the average density of six Reference Spots. **P < 0.01 and ****P < 0.0001 vs. NF cells. ^####^*P* < 0.0001 vs. NF + *Hp* group. **E** ELISA shows Serpin E1 levels in the culture or co-culture supernatants of *H. pylori*, NFs, and AGS cells. Data are presented as means ± SD from three independent experiments. **P < 0.01 and ****P < 0.0001. **F–H** Transwell assays, cytokine detection by the human cytokine array, and Serpin E1 detection by ELISA in Transwell co-culture of *H. pylori*, CAFs, and AGS cells for 7 days. **P < 0.01, ***P < 0.001, and ****P < 0.0001. ^a^ *P* < 0.001 and ^b^ *P* < 0.05 vs. AGS/Medium

A recent report indicated that the expression of Lumican was higher in gastric CAFs than in NFs [29]. Our transcriptomic analysis also confirmed this result. Therefore, the expressions of FAP, Lumican, and α-SMA were used to assess the activation of NFs to CAFs in the direct co-culture of *H. pylori*, NFs, and/or AGS cells, and indirect Transwell co-culture, in which *H. pylori* and/or AGS cells were seeded into the upper chamber and NFs into the lower chamber of the Transwell (0.4-μm pore size). The results showed that, except for the higher expression of FAP and Lumican in CAFs than in NFs, the expression of the two proteins was significantly increased in both direct co-cultured NFs and Transwell co-cultured NFs, especially FAP expression in a time-dependent manner in the direct co-cultures (Figs. 3A, B). Of these, *H. pylori* infection alone had minimal effects on activating NF to CAF phenotype. In addition, no statistical differences in the expression of α-SMA were found between NFs and co-cultured NFs (Additional file 1: Fig. S1). Furthermore, *H. pylori* infection of both AGS cells in the upper chamber and NFs in the lower chamber significantly promoted the migration and invasion of AGS cells (Fig. 3C), suggesting that their interaction enhanced the function of NFs. Nevertheless, there were no significant differences in the migration and invasion of AGS cells between the AGS/Medium and AGS/NF groups or the *Hp* + AGS/Medium and *Hp* + AGS/NFs groups (Fig. 3C), implying that *H. pylori* infection may play a more crucial role in the process.

Next, cytokines were detected in the co-culture supernatants of *H. pylori*, NFs, and AGS cells using a human cytokine array (Fig. 3D). Only cytokines Serpin E1, CCL2, and MIF were detected in the culture supernatants of NFs, while *H. pylori* infection significantly increased the three-cytokines production in the supernatants. After *H. pylori*, NFs, and AGS cells were co-cultured for seven days, ten cytokines, including Serpin E1, MIF, IL-8, IL-6, CXCL11, CXCL12, CXCL1, ICAM-1, CCL5, and CCL2, were detected in the co-culture supernatants, in which Serpin E1 levels were significantly higher than in the co-culture supernatants of *H. pylori* and NFs. ELISA analysis further verified that the Serpin E1 level was progressively increased in the supernatants from NFs to *H. pylori*-infected NFs, and to the co-culture of *H. pylori*, NFs, and AGS cells (Fig. 3E). ELISA analysis also showed that the highest level of Serpin E1 was found in the culture supernatant of CAFs compared to NFs and AGS cells, and *H. pylori* significantly enhanced the Serpin E1 production in *H. pylori*-infected NFs, AGS cells, and CAFs (Fig. 3E).

Next, we constructed the Transwell co-culture system by AGS cells in the upper chamber and CAFs in the lower chamber with or without *H. pylori* infection and found that the migration and invasion ability of AGS cells gradually increased from the co-culture of AGS cells and CAFs to the co-culture of *H. pylori*-infected AGS cells and CAFs or AGS cell and *H. pylori*-infected CAFs, and to the co-culture of *H. pylori*-infected AGS cell and *H. pylori*-infected CAFs (Fig. 3F). Moreover, Serpin E1 was detected with gradually increased levels in the medium of the lower chamber from the AGS/medium group to the *H. pylori* + AGS / medium group or the AGS/CAF group, and to the *H. pylori* + AGS / *H. pylori* + CAF group (Fig. 3G). The same results were obtained from the ELISA assay (Fig. 3H). In addition, we demonstrated that *H. pylori* were present at the cell surface of AGS, CAF, and NF cells after co-incubation with *H. pylori* for seven days (Additional file 1: Fig. S2**)**.

### *H. pylori* and CAFs induce serpin E1 expression in Mongolian gerbil stomach mucosa and human gastric cancer cells

**Fig. 4:**
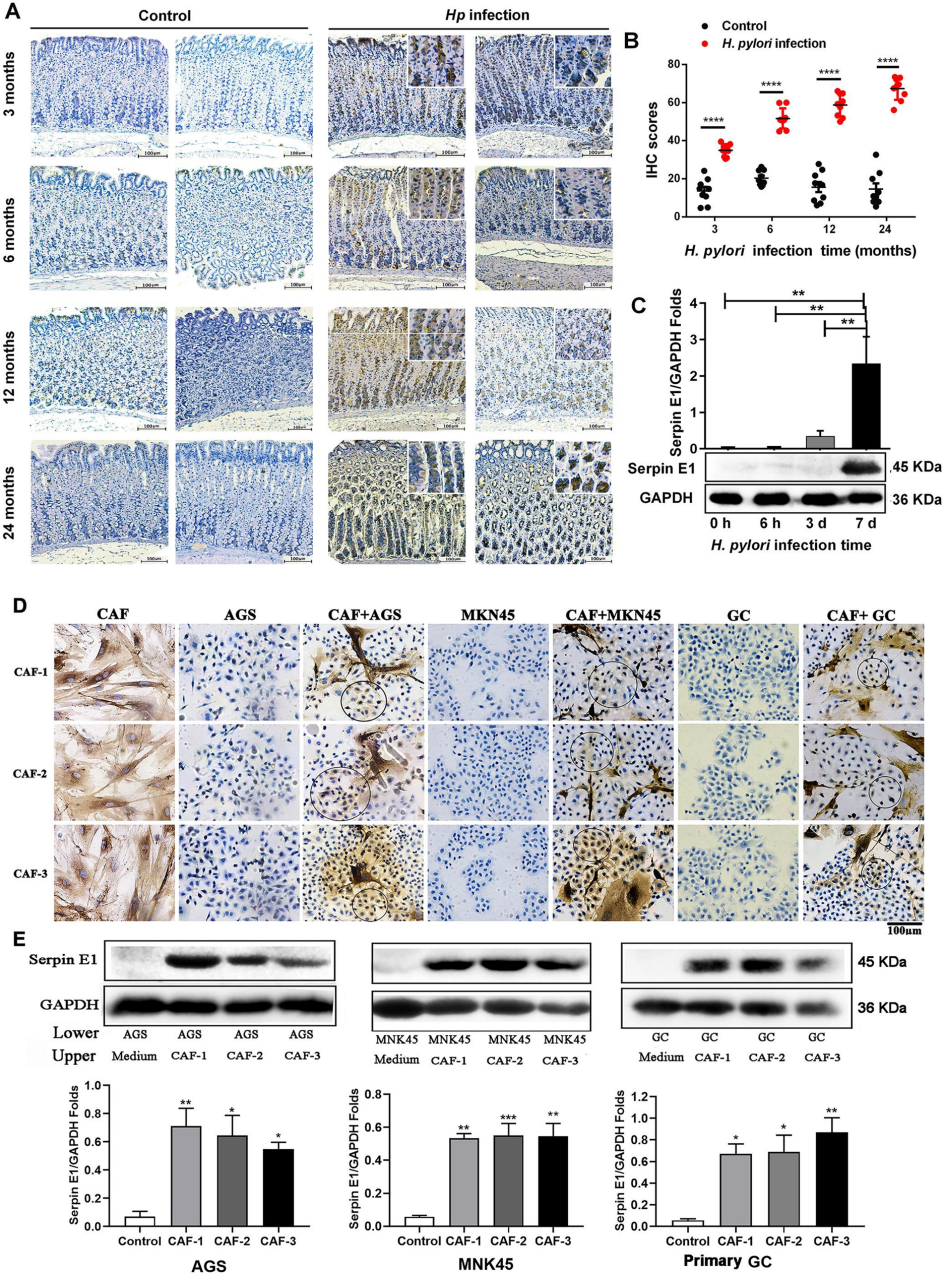
*H. pylori* and CAFs induce Serpin E1 expression in gerbil stomach epithelium and human gastric cancer cells. **A** IHC assay shows positive staining for Serpin E1 in the gastric mucosa of Mongolian gerbils at the indicated times after *H. pylori* infection (n = 3). The gerbils inoculated intragastrically with brain heart infusion serve as the control. Scale bar = 100 μm. **B** Dot-plot graph shows the quantification of Serpin E1 staining using IHC Profiler from Image J software. IHC scores = intensity score × percentage score. Data are presented as means ± SD of nine randomly selected sections from three independent samples. ****P < 0.0001. **C** Western blot analysis for Serpin E1 in AGS cells infected with *H. pylori*. **P < 0.01. **D** IHC staining for Serpin E1 in gastric cancer cells co-cultured with CAFs for 3 days at a 1:1 ratio. CAFs cultured alone for 3 days serve as the positive control. Blue circles indicate positive-staining cells. Scale bar = 100 μm. **E** Western blot analysis for Serpin E1 in gastric cancer cells in the lower chamber after co-culture with CAFs in the upper chamber of the Transwell. The bar graph shows the quantification of Serpin E1 levels. Gastric cancer cells cultured alone serve as the controls. *P < 0.01, **P < 0.01, and ***P < 0.001 vs. control

In our previous study, *H. pylori* NCTC 11637 was used to infect intragastrically Mongolian gerbils for 24 months to establish *H. pylori*-related gastric disease models. In these gerbils, mucosa erosion, atrophic gastritis, intestinal metaplasia, dysplasia, and well-differentiated gastric adenocarcinoma were pathologically observed from 3 to 24 months after *H. pylori* infection [26]. In the current study, Serpin E1 expression was detected in the stomach mucosa of these gerbils by immunohistochemistry (IHC). The results indicated that the positive Serpin E1 staining increased progressively over the infection time from 3 to 24 months, and the scope of the positive staining was also gradually expanded from the upper region to the lower region of gastric epithelial (Fig. 4A, B), suggesting that Serpin E1 expression was associated with pathological progression of gastric cancer caused by *H. pylori* infection in gerbils. In addition, *H. pylori* infection also induced Serpin E1 expression in AGS cells in vitro (Fig. 4C).

Next, AGS, MNK45, and primary gastric cancer (primary GC) cells that did not express Serpin E1 in vitro were co-cultured with three primary CAFs, respectively, and the positive Serpin E1 staining was observed in the cancer cells adjacent to CAFs (Fig. 4D). Furthermore, Serpin E1 protein was highly expressed in three cancer cell lines co-cultured with CAFs added into the upper chamber of the Transwell insert (0.4-µm pore size) (Fig. 4E). Identification of the primary GC cells is shown in Additional file 1: Fig. S3.

### Serpin E1 overexpression promotes the growth, migration, and invasion of gastric cancer cells and inhibits AS_2_O_3_-induced apoptosis in vitro

**Fig. 5:**
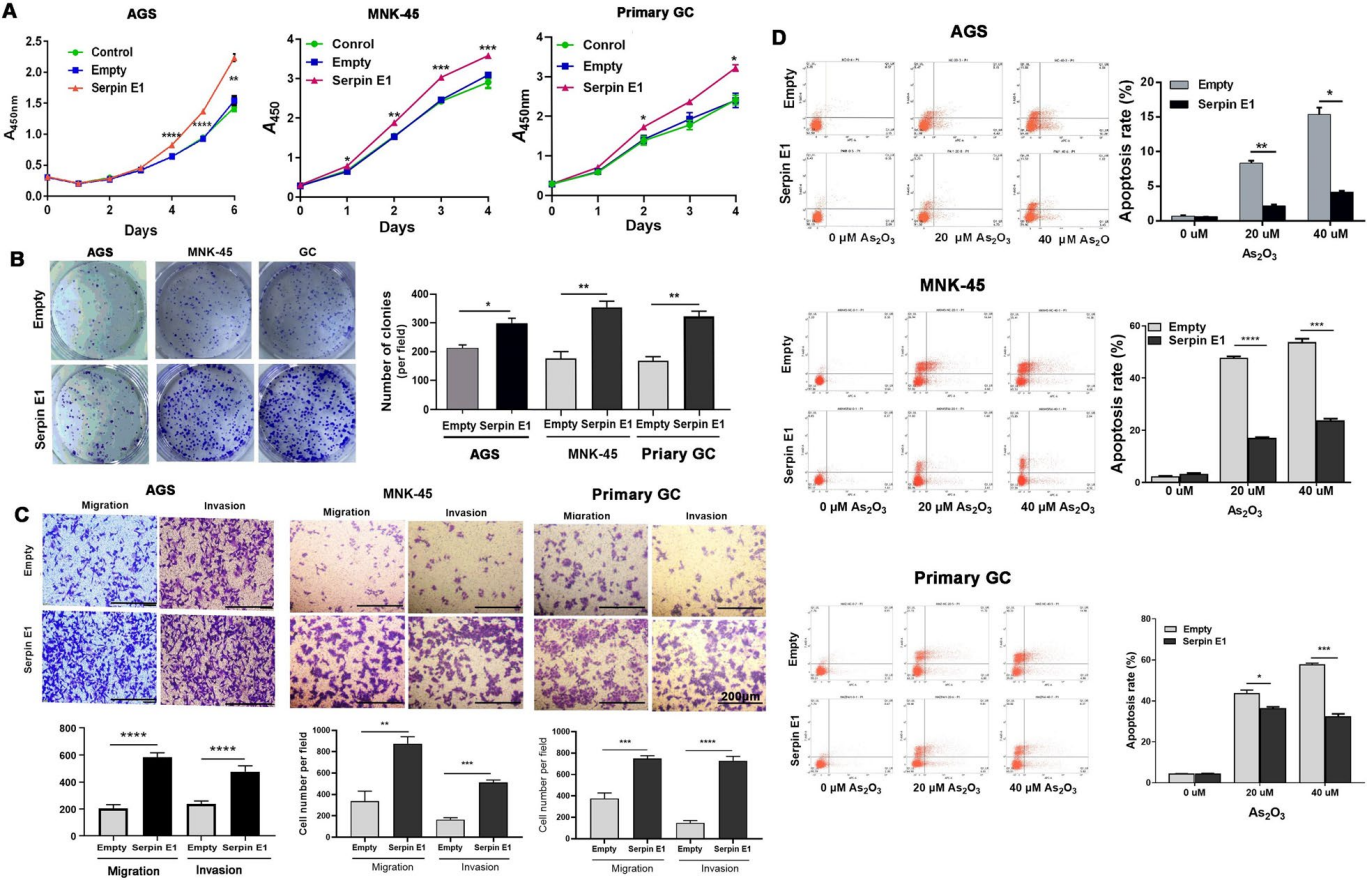
Serpin E1 overexpression promotes the growth, migration, and invasion and inhibits the apoptosis of gastric cancer cells. **A** Growth curve of three gastric cancer cells by a CCK-8 assay upon Serpin E1 overexpression. **P < 0.01 and ****P < 0.0001 vs. Empty. **B** Colony formation assays of three gastric cancer cells upon Serpin E1 overexpression. The bar graph shows the quantitation of the colony number of cancer cells. *P < 0.05 and **P < 0.01. **C** Transwell migration and Matrigel invasion assays of three gastric cancer cells upon Serpin E1 overexpression. The bar graph shows the quantification of migrated or invaded cells. Scale bar = 200 μm. **D** The apoptosis of three gastric cancer cells induced by As_2_O_3_ upon Serpin E1 overexpression was examined by flow cytometry analysis. The bar graph shows the apoptosis rate of cancer cells at the indicated concentration of As_2_O_3_. *P < 0.05, ***P < 0.001, and **** *P* < 0.0001

We established three gastric cancer cell lines, including AGS, MNK45, and primary GC cells, stably overexpressing Serpin E1 and found that Serpin E1 overexpression promoted the malignant phenotype of the three cell lines by significantly promoting the cell growth and colony formation and enhancing the migration and invasion of cancer cells (Fig. 5A–C). Subsequently, we used arsenic trioxide (As_2_O_3_: 20 μΜ and 40 μΜ) to induce cell apoptosis and found that Serpin E1 overexpression inhibited the As_2_O_3_-induced apoptosis of three cancer cell lines in a dose-independent manner (Fig. 5D). These findings revealed an in vitro tumor-promoting effect of Serpin E1 in gastric cancer.

### Serpin E1 overexpression promotes tumor growth in nude mice via inducing angiogenesis

**Fig. 6:**
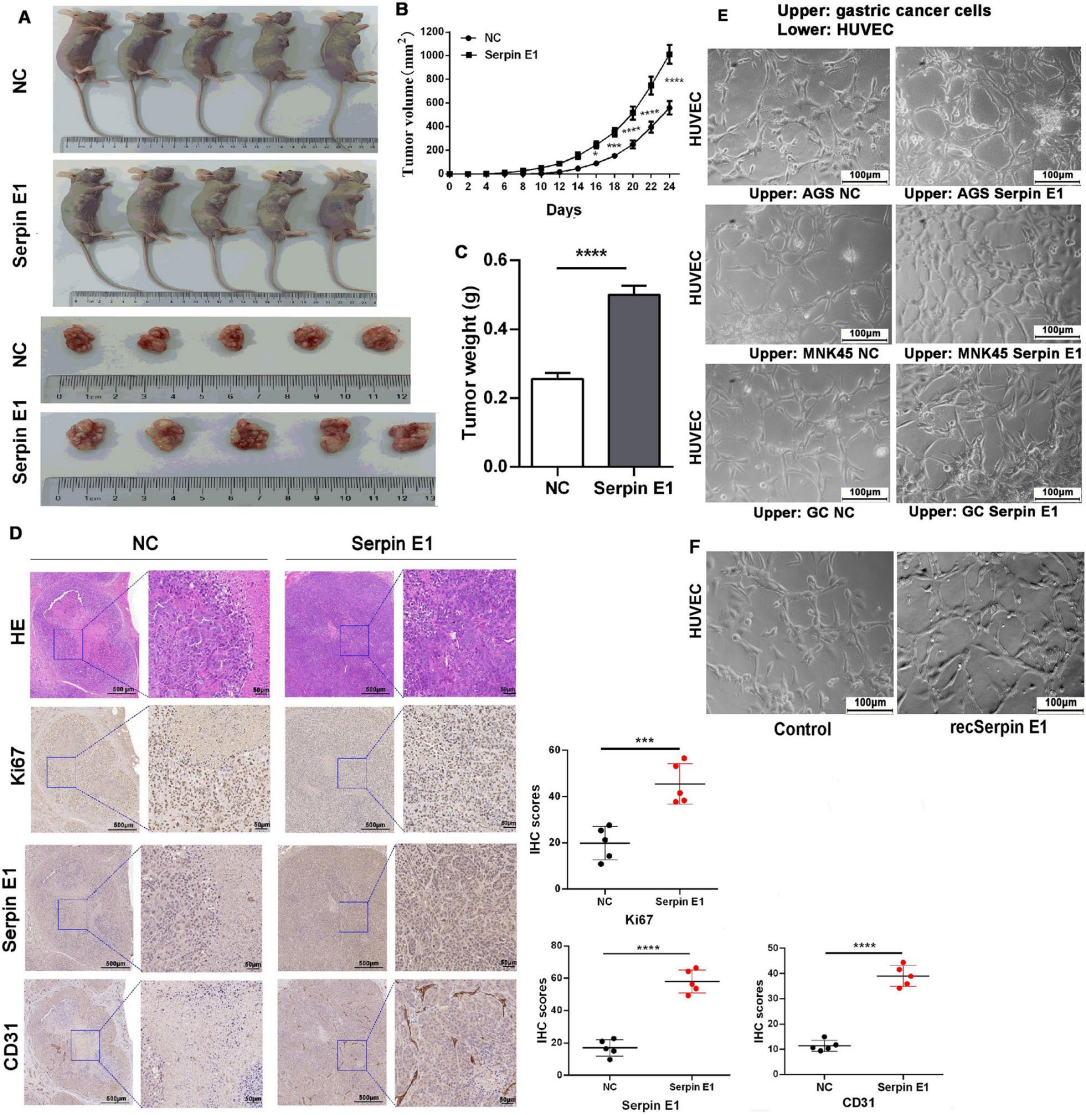
Serpin E1 overexpression promotes the growth of xenograft tumors via inducing neovascularization in nude mice. **A** In vivo tumor formation assay shows that the xenograft tumors with Serpin E1-overexpression display faster growth than control tumors (NC). **B** Growth curve of xenograft tumors by measuring the tumor volume (V). V = 1/2 × length × width^2^ (mm3). * *P* < 0.05, ***P < 0.001, and **** *P* < 0.0001. **C** Weight of xenograft tumors removed from nude mice. **** *P* < 0.0001. **D** HE staining and IHC analysis for Ki 67, Serpin E1, and CD31 in xenograft tumors removed from nude mice. Scale bar = 500 μm. The blue square area is enlarged to the right of each image. Scale bar: 500 μm and 50 μm in the original and enlarged images, respectively. Dot-plot graph shows the quantification of IHC staining for Ki67, Serpin E1, and CD31. IHC scores = intensity score × percentage score. Data are presented as means ± SD from five independent samples. **** *P* < 0.0001. **E** Matrigel tube formation assay of HUVECs co-cultured with Serpin E1-overexpressed gastric cancer cells using the Transwell co-culture system. HUVECs were seeded in the lower chamber and gastric cancer cells in the upper chamber of the Transwell insert. GC represents primary gastric cancer cells. Scale bar = 100 μm. **F** Matrigel tube formation assay of HUVECs treated with human recombinant Serpin E1 (recSerpin E1) at a concentration of 2 ng/ml. Scale bar = 100 μm

The primary GC cells stably overexpressing Serpin E1 were used to establish human tumor xenografts in nude mice subcutaneously. Compared with tumor-bearing control nude mice (NC) in which the tumor cells were transfected with empty vector lentivirus, Serpin E1 overexpression in cancer cells could promote in vivo xenograft tumor formation and growth (Fig. 6A). The volume and weight of the xenograft tumors were significantly increased in the Serpin E1 overexpression group compared to the NC group (Fig. 6B, C). Subsequently, the tumors were removed from nude mice. Hematoxylin and Eosin (HE) staining showed significant necrosis in the center of xenograft tumors in the NC group, but Serpin E1 overexpression in cancer cells can ameliorate the necrosis in the center of the tumor, as shown in the Serpin E1 overexpression group (Fig. 6D). This result was further confirmed by Ki67 staining (Fig. 6D). We next detected CD31 expression, a blood vessel endothelial cell marker, in xenograft tumors and found Serpin E1 could induce new blood vessel formation, especially in the necrotic center of the xenografts, leading to the improvement of ischemia-induced cell necrosis (Fig. 6D). Serpin E1 overexpression and human recombinant Serpin E1 (recSerpin E1) also promoted the tube formation of HUVECs in vitro (Fig. 6E, F). Interestingly, the primary GC cells that did not express Serpin E1 in vitro have positive Serpin E1 staining at low levels in the NC group (Fig. 6D). We speculated that fibroblasts from subcutaneous tissues of nude mice probably induced Serpin E1 expression.

### High expression of serpin E1 and FAP in clinical gastric cancer tissues

**Fig. 7:**
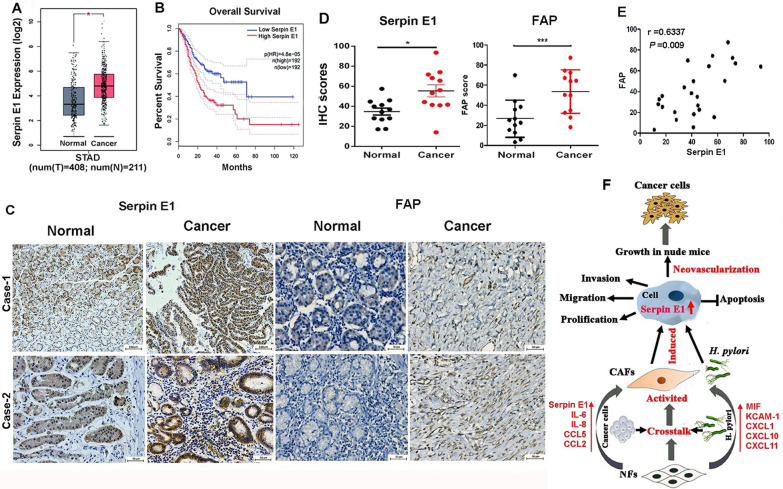
High Serpin E1 expression in gastric cancer tissues from the TCGA-STAD database and patients with gastric cancer. **A** Analysis of the TCGA-STAD dataset, including 408 tumors and 211 normal tissues, shows the upregulation of Serpin E1 mRNA expression in gastric cancer tissues. *P < 0.05. **B** Kaplan–Meier survival curve shows an inverse correlation of Serpin E1 mRNA level with the ten-year overall survival of patients with gastric cancer based on the TCGA-STAD dataset. **C** IHC staining for Serpin E1 and FAP in clinical gastric cancer samples (n = 12). Scale bar: 100 μm for the upper left images and 50 μm for the remaining images. **D** Quantification of IHC staining for Serpin E1 and FAP. IHC scores = intensity score × percentage score. Data are presented as means ± SD from 12 independent samples. *P < 0.05 and *** *P* < 0.001. **E** Spearman correlation analysis between Serpin E1 and FAP expression in 24 clinical gastric tissue samples. **F** A possible mechanism of *H. pylori*-NFs/CAFs-cancer cells crosstalk in gastric tumorigenesis

We downloaded the genomic dataset of human gastric cancer from the TCGA-STAD database, including 408 tumors and 211 normal tissues, and found that the expression of Serpin E1 mRNA in gastric cancer tissues was significantly higher than that in normal tissues (Fig. 7A). Moreover, Serpin E1 mRNA levels were inversely correlated to the ten-year overall survival of patients with gastric cancer (Fig. 7B). Furthermore, we determined the Serpin E1 and FAP expression in 12 clinical gastric cancer tissues and paired para-cancer normal tissues by IHC staining and found that Serpin E1 and FAP were mainly expressed in cancer cells and CAFs, respectively. Moreover, the expression of the two proteins was significantly higher in cancer tissues than in normal tissues (Fig. 7C, D). Correlation analysis showed a high degree of interaction between them (Fig. 7E), suggesting the association between CAFs and Serpin E1 expression in cancer cells. Interestingly, lower Serpin E1 expression was observed in stromal cells. A possible mechanism through which the crosstalk between *H. pylori*, NFs/CAFs, and cancer cells promotes gastric tumorigeneses was illustrated in Fig. 7F.

### Discussion

In this study, we obtained 161 DEGs between matched gastric NFs and CAFs and demonstrated that the interplay of *H. pylori*, fibroblasts, and gastric cancer cells enhanced the conversion of NFs to CAFs via cell-to-bacterium and cell-to-cell interaction and various cytokine production. We also revealed that *H. pylori* and CAFs cooperatively induced Serpin E1 expression in cancer cells to promote gastric cancer tumorigenesis and progression in vitro and in vivo.

CAFs have considerable heterogeneity in their function and marker expression [22]. The molecular alterations, including transcriptome, proteome, and metabolome, have occurred during the transition from NFs to CAFs in multiple solid tumors such as oral squamous cell cancer [30], breast cancer [31], and colon cancer [32]. By RNA-seq, we revealed the differences in transcriptomic profiles between primary CAFs and NFs isolated from gastric adenocarcinoma tissues and adjacent normal tissues, respectively. Among the DEGs, we confirm that FAP and lumican proteins, two characteristic markers of CAFs, are highly expressed in CAFs compared to NFs, but there is no difference in α-SMA expression, a feature of myofibroblasts present in some types of cancers, between CAFs and NFs by our RNA-seq. The result is partly supported by previous reports, which showed that α-SMA^low^CAFs are present in breast and pancreatic cancer and grouped in inflammatory CAFs [33, 34]. Two articles also showed that CAFs-derived FAP or lumican promoted gastric cancer progression, and their expression was related to poor clinical outcomes [26, 35]. The inflammatory CAFs are characterized by a secretory and inflammatory phenotype. This type of CAFs can secret cytokines IL6 and IL11 [18, 36]. Likewise, IL18 and IL33 mRNA are highly expressed in CAFs by our RNA-seq analysis. These data strongly support that gastric adenocarcinoma consists predominantly of inflammatory CAFs with α-SMA^low^FAP^high^ lumican^high^ markers, although the myofibroblasts with α-SMA^high^CAFs were found in scirrhous gastric cancer [37, 38].

Our study indicates that the interplay of *H. pylori*, fibroblasts, and cancer cells induces inflammatory cytokine release to promote the transition of NFs to CAFs by showing an increased expression of FAP and Lumican in NFs co-culture with *H. pylori* and gastric cancer cells. This interplay further enhances the NFs-mediated migration and invasion of AGS cells. The direct interactions of *H. pylori*, NFs, and cancer cells have the strongest ability to drive the transition of NFs to CAFs through ten cytokine productions, including Serpin E1, IL6, IL8, etc. Further, *H. pylori* infection significantly enhances NFs- and CAFs-mediated migration and invasion of AGS cells. These findings reveal that the *H. pylori*-induced inflammatory microenvironment is crucial for fibroblasts activation in gastric cancer. However, most of the ten cytokines have not been intensively investigated in gastric diseases. In a recent study, Shen et al. reported that *H. pylori* infection activates stomach fibroblasts and increases vascular adhesion molecule 1 expression in CAFs, resulting in tumor progression in gastric cancer [39]. *H. pylori* infection-induced differentiation of rat stomach fibroblasts into CAFs in vitro was also reported [40].

Serpin E1 was significantly upregulated in gastric cancer tissues and was correlated with poor prognosis of gastric cancer patients by microarray data analysis and bioinformatics [41–43]. Overexpression of Serpin E1 was also related to aggressive lymph node metastasis in advanced gastric cancer via DNA microarray analysis and validation by RT-qPCR and tissue-microarray, but gastric adenocarcinoma with T4N0 stage showed negative Serpin E1 staining [44]. Such contradictory results point to the need for more experimental evidence. Moreover, the reason for the high expression of Serpin E1 in gastric cancer cells remains unknown. In the current study, we reveal, for the first time, that long-term *H. pylori* infection stimulates the progressive increase of Serpin E1 expression in gerbil gastric epithelium infected with *H. pylori* for two years. CAFs also induce Serpin E1 expression in AGS, MNK45, and primary GC cells via the direct interaction and paracrine manner. These findings strongly support the role of *H. pylori*- and CAFs-induced Serpin E1 expression in gastric carcinogenesis. A study by Kenny et al. observed the increased expression of Serpin E1 in the gastric epithelial cells of *H. pylori*-positive patients yet [45], which further supports our results.

Serpin E1 promotion of cancer progression has been studied in multiple solid tumors, including pancreas, prostate, liver, and lung cancer [46], but little in stomach cancer, particularly the involvement of *H. pylori*. In the current study, the gastric cancer-promoting roles of Serpin E1 in vitro and in vivo and in human gastric cancer tissues were observed. Our study clearly shows that Serpin E1 can facilitate the growth, migration, and invasion of gastric cancer cells in vitro and tumor formation and growth in nude mice. Furthermore, the tumor-promoting effect of serpin E1 is achieved by inhibiting cell apoptosis in vitro and inducing new blood vessel formation in the necrotic region in the center of xenografts in vivo. These observations support the pro-angiogenesis and anti-apoptosis functions of Serpin E1 established in other tumors [47–49]. A recent study by Chen et al. showed that Serpin E1 upregulated VEGF expression in gastric cancer cells [50]. Also, Teng et al. found that lncRNA NKX2-1-AS1 promotes angiogenesis via upregulating Serpin E1 expression leading to VEGFR2 pathway activation in gastric cancer cells [51]. Thereby, VEGF/VEGFR2 signaling may be the contribution of Serpin E1-induced angiogenesis. Finally, we found that Serpin E1 and FAP were significantly more highly expressed in cancer cells and CAFs of gastric cancer tissues, respectively, and there was a significantly positive interaction between their expression, further suggesting the association between CAFs and Serpin E1 expression in cancer cells.

In conclusion, our study reveals that the crosstalk of *H. pylori*, NFs, and cancer cells promotes the activation of NFs to CAFs via cytokines release, especially Serpin E1. *H. pylori* and CAFs together induce gastric epithelial/cancer cells to express Serpin E1, ultimately promoting the tumorigenesis and progression of gastric cancer. Targeting Serpin E1 will provide a promising therapeutic strategy for gastric cancer by disrupting the interaction between *H. pylori*, CAFs, and gastric cancer cells, which will become an attractive target for future research.

## Supplementary Information

**Additional file 1: Table S1.** Human Cytokine Array List. **Table S2.** Characteristics of 161 differentially expressed genes between CAFs and NFs. **Figure S1.** Western blot shows α-SMA expression in the direct and Transwell (indirect) co-culture of *H. pylori*, NFs, and AGS cells for the indicated time at an MOI of 50. The bar graph shows the quantitation of α-SMA levels. **Figure S2.** *H. pylori* were present at the cell surface of AGS, CAF, and NF cells infected with *H. pylori* for 7 days via immunofluorescence staining (**A**) and Western blot analysis for urease B (**B**), a subunit of urease enzyme generated by *H. pylori*. Free *H. pylori* were removed via washing with PBS at 6 h after infection. **Figure S3.** Primary gastric cancer cells isolated from gastric cancer tissues were identified by CK-18 and CEA immunohistochemistry staining, soft agar colony formation, and tumor formation in nude mice.

## Data Availability

The dataset supporting the conclusions of this article is included within the article and its additional files.

## Abbreviations

As_2_O_3_: Arsenic trioxide
α-SMA: α-Smooth muscle actin
*H. pylori*: *Helicobacter pylori*
CAFs: Cancer-associated fibroblasts
CagA: Cytotoxin-associated gene A
DEGs: Differentially expressed genes
FAP: Fibroblast-activating protein
FSP1: Fibroblast specific protein 1
GC: Gastric cancer
GO: Gene ontology
GSEA: Gene set enrichment analysis
IARC: International agency for research on cancer
ICC: Immunocytochemistry
IF: Immunofluorescence
IHC: Immunohistochemistry
IL: Interleukine
LUM: Lumican
KEGG: Kyoto encyclopedia of genes and genomes
NFs: Normal fibroblasts
PAI-1: Plasminogen activator inhibitor-1
PPI: Protein–protein interaction
SPARC: Secreted protein acidic and rich in cysteine
RNA-seq: RNA-sequencing
RT-qPCR: Quantitative reverse transcription PCR
TCGA-STAD: The cancer genome atlas-stomach adenocarcinoma
TME: Tumor microenvironment
