# Supplementary material for: Interplay of Helicobacter pylori, fibroblasts, and cancer cells induces fibroblast activation and serpin E1 expression by cancer cells to promote gastric tumorigenesis
Source: J Transl Med. 2022 Jul 21;20:322. doi: 10.1186/s12967-022-03537-x (PMC9306099; doi:10.1186/s12967-022-03537-x)
Supplement: Supplementary file 1 — Additional file 1: Table S1. Human Cytokine Array List. Table S2. Characteristics of 161 differentially expressed genes between CAFs and NFs. Figure S1. Western blot shows α-SMA expression in the direct and Transwell (indirect) co-culture of H. pylori, NFs, and AGS cells for the indicated time at an MOI of 50. The bar graph shows the quantitation of α-SMA levels. Figure S2. H. pylori were present at the cell surface of AGS, CAF, and NF cells infected with H. pylori for 7 days via immunofluorescence staining (A) and Western blot analysis for urease B (B), a subunit of urease enzyme generated by H. pylori. Free H. pylori were removed via washing with PBS at 6 h after infection. Figure S3. Primary gastric cancer cells isolated from gastric cancer tissues were identified by CK-18 and CEA immunohistochemistry staining, soft agar colony formation, and tumor formation in nude mice. [file 12967_2022_3537_MOESM1_ESM.pdf]

Figure S1. Western blot shows α-SMA expression in the direct and Transwell (indirect) co-culture of *H. pylori*, NFs, and AGS cells for 6 h, 72 h, and 7 days at a multiplicity of infection (MOI) of 50. The bar graph shows the quantitation of α-SMA levels.

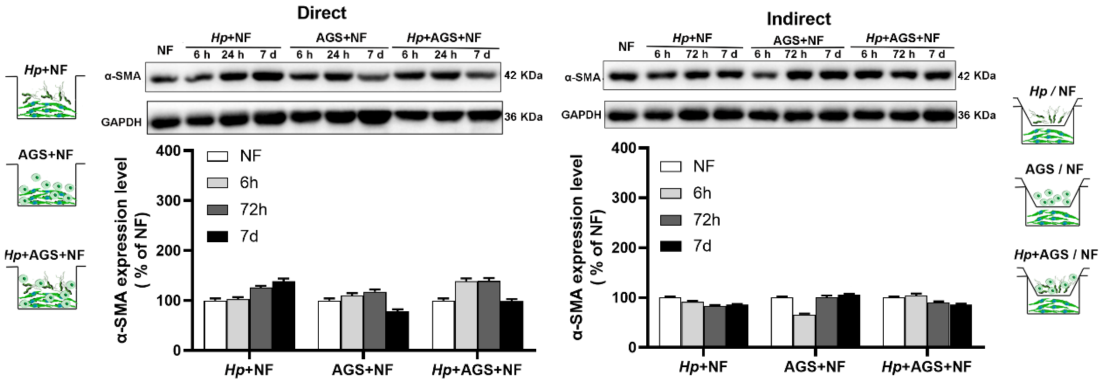

Figure S2. *H. pylori* were present at the cell surface of AGS, CAF, and NF cells infected with *H. pylori* for 7 days via confocal immunofluorescence (**A**) and the expression of urease B (**B**), a subunit of urease enzyme generated by *H. pylori*. Free *H. pylori* were removed via washing with PBS at 6 hours after infection.

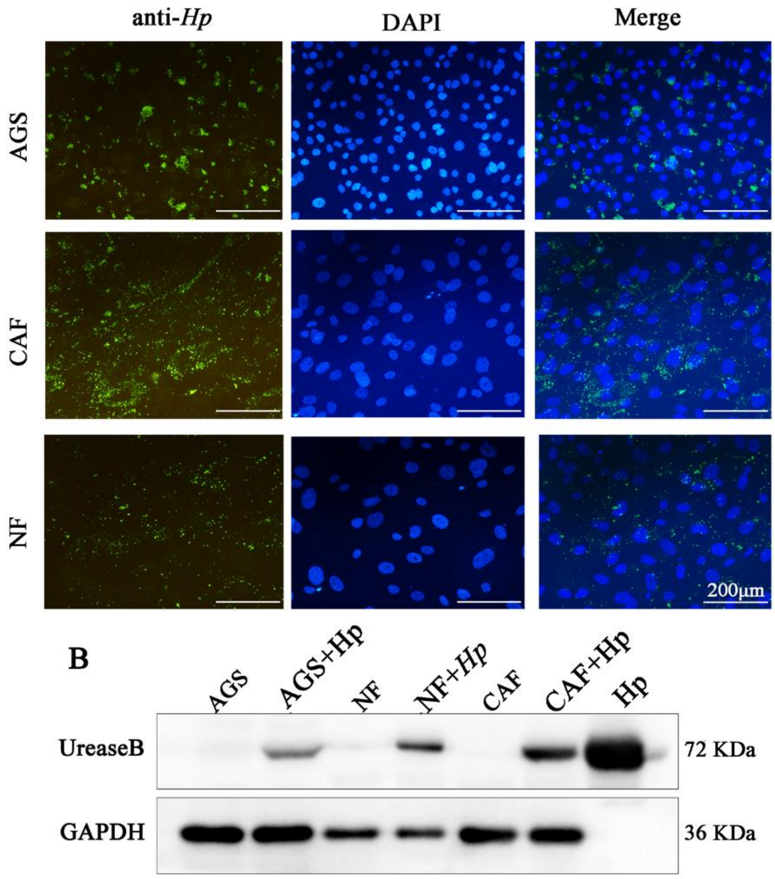

Figure S3. Primary gastric cancer cells were isolated from gastric cancer tissues and

identified by CK-18 and CEA immunohistochemistry staining, soft agar colony formation, and tumor formation in nude mice.

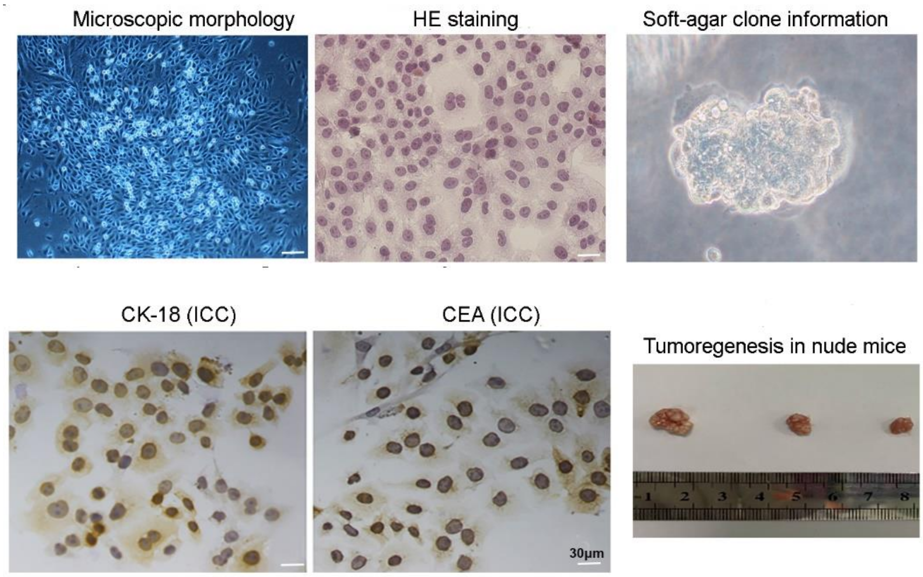

Table S1. Human Cytokine Array List

Human Cytokine Array Coordinates

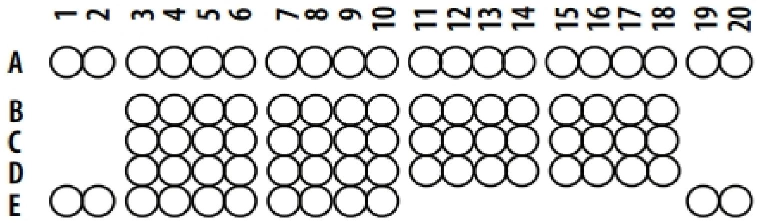

Coordinate	Target/Control	Entrez Gene ID	Alternate Nomenclature
**E19, E20**	Negative Control	N/A	-
**A1, A2**	Reference Spots	N/A	-
**A19, A20**	Reference Spots	N/A	-
**E1, E2**	Reference Spots	N/A	-
**A3, A4**	CCL1	6346	P500, SCYA1, SCYA2, TCA-3
**A5, A6**	CCL2	6347	MCAF
**A7, A8**	MIP-1α	6348/6351	CCL3/CCL4
**A9, A10**	CCL5	6352	-
**A11, A12**	CD40 Ligand	959	CD154, CD40LG, gp39, TRAP
**A13, A14**	Component C5	727	C5/C5a
**A15, A16**	CXCL1	2919	CINC-1, KC
**A17, A18**	CXCL10	3627	CRG-2
**B3, B4**	CXCL11	6373	β-R1, H174
**B5, B6**	CXCL12	6387	PBSF
**B7, B8**	G-CSF	1440	CSFβ, CSF-3
**B9, B10**	GM-CSF	1437	CSFα, CSF-2
**B11, B12**	ICAM-1	3383	-
**B13, B14**	IFN-γ	3458	Type II IFN
**B15, B16**	IL-1α	3552	-
**B17, B18**	IL-1β	3553	-
**C3, C4**	IL-1ra	3557	-
**C5, C6**	IL-2	3558	TCGF
**C7, C8**	IL-4	3565	BCDF, BSF1
**C9, C10**	IL-5	3567	-
**C11, C12**	IL-6	3569	BSF-2
**C13, C14**	IL-8	3576	CXCL8, GCP1, NAP1
**C15, C16**	IL-10	3586	CSIF
**C17, C18**	IL-12 p70	3592/3593	CLMF p35
**D3, D4**	IL-13	3596	-
**D5, D6**	IL-16	3603	LCF
**D7, D8**	IL-17A	3605	CTLA-8
**D9, D10**	IL-17E	64806	IL-25
**D11, D12**	IL-18	3606	IGIF
**D13, D14**	IL-21	59067	-
**D15, D16**	IL-27	246778	IL-27 A
**D17, D18**	IL-32α	9235	-
**E3, E4**	MIF	4282	GIF, DER6
**E5, E6**	Serpin E1	5054	Nexin, PLANH1
**E7, E8**	TNF-α	7124	TNFSF1A
**E9, E10**	TREM-1	54210	CD354

Table S2. Characteristics of 161 differentially expressed genes between CAFs and NFs

N	Gene Symbol	Log2FC (CAF/NF) mean	pvalue	FDR	Regulation
1	IL33	4.86	0.0270	0.0350	UP
2	ELN	3.97	0.0301	0.0370	UP
3	PDK4	3.64	0.0217	0.0315	UP
4	GALNT15	3.25	0.0272	0.0350	UP
5	APCDD1L	3.04	0.0003	0.0095	UP
6	LXN	3.04	0.0262	0.0346	UP
7	KYNU	2.98	0.0060	0.0179	UP
8	PCDH7	2.93	0.0125	0.0234	UP
9	ACKR3	2.91	0.0295	0.0370	UP
10	AOC3	2.89	0.0333	0.0394	UP
11	PTGFR	2.82	0.0289	0.0366	UP
12	LUM	2.8	0.0020	0.0112	UP
13	AKR1C2	2.79	0.0338	0.0397	UP
14	MGP	2.76	0.0443	0.0466	UP
15	COL3A1	2.68	0.0426	0.0457	UP
16	DPP4	2.65	0.0498	0.0498	UP
17	ADAM12	2.63	0.0165	0.0268	UP
18	MT1M	2.59	0.0260	0.0346	UP
19	S100A4	2.53	0.0354	0.0401	UP
20	AKR1C1	2.51	0.0254	0.0346	UP
21	ISLR	2.41	0.0019	0.0112	UP
22	LRRK2	2.36	0.0387	0.0427	UP
23	CPE	2.21	0.0184	0.0285	UP
24	SELENOP	2.21	0.0430	0.0458	UP
25	SPARC	2.21	0.0132	0.0242	UP
26	MEGF9	2.15	0.0058	0.0177	UP
27	ABHD5	2.11	0.0014	0.0112	UP
28	INSIG1	2.08	0.0096	0.0212	UP
29	SSH2	2.00	0.0206	0.0307	UP
30	IL18	1.99	0.0224	0.0322	UP
31	AGTR1	1.97	0.0087	0.0205	UP
32	CHRM2	1.94	0.0474	0.0480	UP
33	SAA1	1.92	0.0114	0.0231	UP
34	FBN1	1.91	0.0079	0.0203	UP
35	PDE1A	1.91	0.0210	0.0310	UP
36	PTN	1.9	0.0028	0.0124	UP
37	CRYBG3	1.88	0.0139	0.0246	UP
38	PLA2R1	1.82	0.0085	0.0205	UP
39	VCAN	1.81	0.0088	0.0205	UP
40	COL4A5	1.79	0.0310	0.0378	UP
41	KCNK1	1.76	0.0008	0.0112	UP
42	ATL1	1.70	0.0001	0.0061	UP
43	TNFAIP8L3	1.63	0.0024	0.0117	UP
44	FAP	1.62	0.0152	0.0255	UP
45	SLC4A4	1.59	0.0470	0.0479	UP
46	ATP2B4	1.52	0.0001	0.0061	UP

N	Gene Symbol	Log2FC (CAF/NF) mean	pvalue	FDR	Regulation
47	HS2ST1	1.46	0.0058	0.0177	UP
48	TSHZ1	1.46	0.0049	0.0172	UP
49	C4orf33	1.43	0.0236	0.0336	UP
50	ZNF175	1.42	0.0019	0.0112	UP
51	TCF4	1.39	0.0213	0.0312	UP
52	TMEM154	1.32	0.0241	0.0340	UP
53	POGLUT2	1.29	0.0326	0.0389	UP
54	RNF168	1.24	0.0079	0.0203	UP
55	SOCS5	1.24	0.0025	0.0117	UP
56	TMEM50B	1.24	0.0008	0.0112	UP
57	DOP1A	1.23	0.0002	0.0074	UP
58	ZHX1	1.22	0.0344	0.0397	UP
59	IDI1	1.20	0.0257	0.0346	UP
60	LSM11	1.17	0.0145	0.0247	UP
61	KANK1	1.16	0.0346	0.0397	UP
62	KDELR3	1.12	0.0055	0.0175	UP
63	SUPV3L1	-1.14	0.0047	0.0172	DOWN
64	ROBO3	-1.15	0.0007	0.0112	DOWN
65	TMEM54	-1.16	0.0011	0.0112	DOWN
66	USE1	-1.20	0.0054	0.0175	DOWN
67	P3H2	-1.24	0.0182	0.0284	DOWN
68	SLC25A4	-1.26	0.0142	0.0247	DOWN
69	FUOM	-1.28	0.0010	0.0112	DOWN

N	Gene Symbol	Log2FC (CAF/NF) mean	pvalue	FDR	Regulation
70	DDX10	-1.31	0.0170	0.0274	DOWN
71	KNDC1	-1.32	0.0300	0.0370	DOWN
72	PTP4A3	-1.33	0.0259	0.0346	DOWN
73	SMIM1	-1.35	0.0050	0.0172	DOWN
74	PIP5KL1	-1.36	0.0086	0.0205	DOWN
75	CYSTM1	-1.40	0.0014	0.0112	DOWN
76	SLC35F6	-1.43	0.0266	0.0348	DOWN
77	SLC1A4	-1.49	0.0005	0.0109	DOWN
78	AZIN2	-1.50	0.0014	0.0112	DOWN
79	MTSS2	-1.54	0.0013	0.0112	DOWN
80	SPATA24	-1.54	0.0071	0.0193	DOWN
81	LZTS3	-1.55	0.0016	0.0112	DOWN
82	MCRIP2	-1.57	0.0108	0.0229	DOWN
83	UCHL1	-1.57	0.0434	0.0460	DOWN
84	TSTD1	-1.59	0.0297	0.0370	DOWN
85	ATP2B1	-1.61	0.0493	0.0496	DOWN
86	ERN1	-1.62	0.0024	0.0117	DOWN
87	BRSK1	-1.63	0.0069	0.0193	DOWN
88	EMC9	-1.63	0.0025	0.0117	DOWN
89	FGF21	-1.63	0.0448	0.0468	DOWN
90	DMWD	-1.65	0.0019	0.0112	DOWN
91	HES7	-1.65	0.0089	0.0206	DOWN
92	APBA2	-1.68	0.0115	0.0231	DOWN

N	Gene Symbol	Log2FC (CAF/NF) mean	pvalue	FDR	Regulation
93	TMEM156	-1.68	0.0163	0.0268	DOWN
94	H1-0	-1.70	0.0049	0.0172	DOWN
95	SIX2	-1.71	0.0055	0.0175	DOWN
96	TP53	-1.71	0.0163	0.0268	DOWN
97	AIFM2	-1.75	0.0017	0.0112	DOWN
98	BCL2L1	-1.75	0.0016	0.0112	DOWN
99	IDNK	-1.75	0.0038	0.0161	DOWN
100	RRAD	-1.75	0.0011	0.0112	DOWN
101	SNTB1	-1.76	0.0255	0.0346	DOWN
102	DDIT3	-1.77	0.0129	0.0239	DOWN
103	RAB33A	-1.77	0.0020	0.0112	DOWN
104	GADD45A	-1.83	0.0383	0.0427	DOWN
105	GSTO2	-1.84	0.0203	0.0305	DOWN
106	REEP2	-1.84	0.0115	0.0231	DOWN
107	CDKN3	-1.85	0.0470	0.0479	DOWN
108	ETV5	-1.86	0.0074	0.0196	DOWN
109	ITPR1	-1.86	0.0244	0.0342	DOWN
110	LYL1	-1.86	0.0122	0.0233	DOWN
111	DNAJA4	-1.88	0.0023	0.0117	DOWN
112	UNC5B	-1.88	0.0048	0.0172	DOWN
113	CYB5R2	-1.89	0.0279	0.0357	DOWN
114	PECR	-1.91	0.0119	0.0233	DOWN
115	CTPS1	-1.93	0.0316	0.0383	DOWN

N	Gene Symbol	Log2FC (CAF/NF) mean	pvalue	FDR	Regulation
116	FAM24B	-1.94	0.0120	0.0233	DOWN
117	DLL3	-1.97	0.0006	0.0112	DOWN
118	TRIB3	-1.99	0.0146	0.0247	DOWN
119	CLDN7	-2.02	0.0134	0.0242	DOWN
120	SDSL	-2.06	0.0027	0.0124	DOWN
121	UBE2S	-2.06	0.0088	0.0205	DOWN
122	AJUBA	-2.14	0.0412	0.0445	DOWN
123	PODXL2	-2.14	0.0123	0.0233	DOWN
124	MROH8	-2.15	0.0018	0.0112	DOWN
125	CALHM6	-2.17	0.0387	0.0427	DOWN
126	ZP3	-2.17	0.0261	0.0346	DOWN
127	KCNG1	-2.20	0.0044	0.0167	DOWN
128	PRR11	-2.20	0.0468	0.0479	DOWN
129	NUDT14	-2.22	0.0117	0.0233	DOWN
130	RAB39B	-2.22	0.0095	0.0212	DOWN
131	SH3BGR	-2.22	0.0397	0.0435	DOWN
132	SLC7A5	-2.22	0.0092	0.0208	DOWN
133	TMEM255B	-2.30	0.0402	0.0437	DOWN
134	PLK1	-2.32	0.0459	0.0477	DOWN
135	DPYSL4	-2.33	0.0348	0.0397	DOWN
136	FRY	-2.33	0.0081	0.0203	DOWN
137	HES6	-2.37	0.0012	0.0112	DOWN
138	RASIP1	-2.38	0.0073	0.0195	DOWN

N	Gene Symbol	Log2FC (CAF/NF) mean	pvalue	FDR	Regulation
139	C2CD3	-2.48	0.0041	0.0166	DOWN
140	MFSD13A	-2.52	0.0001	0.0061	DOWN
141	CHST7	-2.54	0.0112	0.0231	DOWN
142	CCL2	-2.56	0.0374	0.0421	DOWN
143	MKX	-2.56	0.0143	0.0247	DOWN
144	C19orf81	-2.64	0.0189	0.0290	DOWN
145	CCR10	-2.68	0.0174	0.0276	DOWN
146	CDH4	-2.75	0.0175	0.0276	DOWN
147	PPME1	-2.79	0.0042	0.0166	DOWN
148	TPD52L1	-2.97	0.0137	0.0245	DOWN
149	OSCAR	-3.00	0.0104	0.0226	DOWN
150	SPX	-3.03	0.0199	0.0302	DOWN
151	NKX3-2	-3.07	0.0040	0.0163	DOWN
152	CHST2	-3.25	0.0106	0.0228	DOWN
153	CYP3A7	-3.39	0.0065	0.0190	DOWN
154	ATP1A2	-3.46	0.0323	0.0388	DOWN
155	ATF3	-3.47	0.0055	0.0175	DOWN
156	GDF15	-3.50	0.0004	0.0095	DOWN
157	B3GALT2	-3.52	0.0346	0.0397	DOWN
158	BEX1	-3.53	0.0067	0.0193	DOWN
159	C1orf115	-3.84	0.0028	0.0124	DOWN
160	UCP2	-4.20	0.0070	0.0193	DOWN
161	BEX2	-4.96	0.0011	0.0112	DOWN
